# Synthesis, Solid State Structure, and Cytotoxic Activity of a Complex Dimer of Yttrium with Anthranilic Acid against Cancer Cells

**DOI:** 10.1007/s12011-022-03545-4

**Published:** 2023-01-05

**Authors:** Amna S. A. Zidan, Ahmed B. M. Ibrahim, Aref A. M. Aly, Hanan K. Mosbah, Peter Mayer, Saber H. Saber

**Affiliations:** 1 Department of Chemistry, Faculty of Science, Assiut University, Assiut, 71516 Egypt; 2 Department Chemie, Ludwig-Maximilians-Universität München, Butenandtstr. 5-13, Haus, D 81377 München, Germany; 3 Laboratory of Molecular Cell Biology, Department of Zoology, Faculty of Science, Assiut University, Assiut, 71516 Egypt

**Keywords:** Neutral ligands, Zwitter ions, Rare earth elements, XRD, Cytotoxicity, Cancer

## Abstract

This paper presents the synthesis and isolation of a new binuclear complex of yttrium with anthranilic acid (HA). The complex [Y_2_(HA)_6_(H_2_O)_4_] Cl_6_.2C_2_H_5_OH (**C1**) was obtained as single crystals that its X-ray analysis revealed its triclinic P-1 space group in addition to anti-prismatic geometry around each of the yttrium ions. In the complex, the anthranilic acid ligands are bidentate, zwitter ionic and neutral, and the yttrium ions’ charge is only compensated by six chloride ions. The cytotoxicity of this complex against human breast cancer MDA-MB-231 cells, prostate cancer PC-3 cells, and bladder cancer T-24 cells was evaluated. This yttrium complex displayed more cytotoxic activity against the bladder cancer cells with an IC_50_ value of 307.7 μg/ml (223 μM). On the other hand, the activities of complex **C1** against the MDA-MB-231 and PC-3 cells were less significant respectively with IC_50_ values of 1097 μg/ml (796 μM) and 921 μg/ml (669 μM).

## Introduction

The old age history of cancer has recently been proved by the discovery of this disease in the remnants of a more than 4,200 years old Egyptian woman [1]. In 2021, the American Cancer Society estimated new cancer cases and related deaths of about 1,898,160 and 608,570 people in the USA (5200 new cases and 1667 deaths each day) [2] and a dramatic increment in this death rate is expected due to the occurrence of over 200 types of cancer that the early discovery of many types of them is very difficult [3]. Breast cancer is the primary reason for death by neoplasia among women in industrialized countries, as it represents nearly 25% of non-accidental deaths of women 35–54 years of age [3]. The breast cancer frequency was reported to increase up to menopause and subsequently, it continues to rise but at a slower rate [4, 5]. Prostate cancer is the most prevalent cancer in men worldwide and is only after lung cancer as a cause of cancer-related deaths in men [6]; the primary obstacle in approaching feasible therapeutics for prostate cancer chemotherapy is the lack of targeted delivery to the prostate [7, 8]. Bladder cancer is a common malignant tumor in the urogenital system estimated to infect about 430,000 people worldwide in 2012 [9]. This type of cancer is usually treated with tumor resection, but the recurrence rate of bladder cancer is still very high [10].

The epidemic threat of cancer has urged several research groups to the rapid approaching modern-day cancer diagnostic and therapeutic agents [11–16]. After the clinical success of cisplatin (cis-dichlorodiammineplatinum (II)), metal-based chemotherapeutics including several metal complexes and organometallics have been nominated as next-generation anticancer drugs [12–15], but complexes based on rare earth metal ions have not been much investigated for their anticancer activities [11, 16]. Acylhydrazone-based complexes with Ce (III) and Sm (III) have displayed significant anti-tumor activity against human colorectal cancer (lovo), human pancreatic cancer (PATU8988), and human gastric cancer (SGC7901) cell lines [17]. Andiappan et al. [18] studied the *in vitro* cytotoxic behavior of Pr (III) and Yb (III) complexes against cervical (HeLa) and breast (MCF-7) cancer cell lines. Zaho et al. [19] demonstrated *in vitro* antitumor activities of La (III) complexes against human MDA-MB-435 (galactophore), HL-60 (leukocytoma), PC-3MIE8 (prostate), BGC-823 (stomach), and HeLa (cervical) cancer cells.

Yttrium is a congeneric element with lanthanides and has been biologically incorporated into ibritumomab tiuxetan (Zevalin) which is an FDA-approved effective drug for treating B-cell non-Hodgkin’s lymphoma [20]. However, yttrium is biologically utilized the most in its radioactive form “yttrium-90” in immuno-radio therapy against various cancers including lymphoma, leukemia, ovarian, liver, colorectal, pancreatic, and bone cancers [21]. This is through targeting the cancer cells with a specific monoclonal antibody to facilitate the binding between the isotope and the cancer cells and subsequently killing the cancer cells through intense β-radiation [21]. In this paper, we describe the synthesis and crystal structure of a new anthranilic acid complex with Y (III) as a rare earth metal ion and explore the cytotoxic effect of this complex against three human cancer cell lines (MDA-MB-231 breast, PC-3 prostate, and T-24 bladder cancer cells). The selection of anthranilic acid as the ligand in this research is due to interesting biological activities reported for the acid itself and its derivatives [22]. The acid itself contributes to the biochemical synthesis of tryptophan which is vital in medicinal and biological aspects, while several substituted anthranilic acids have been reported to possess high antibacterial, anti-inflammatory, anti-malarial and antineoplastic activities [23–26].

## Experimental

### Materials and Physical Measurements

The syntheses included yttrium chloride hexahydrate and anthranilic acid (HA) both purchased from Sigma-Aldrich in addition to high-purity ethanol. Elemental data for carbon, hydrogen, and nitrogen in complex **C1** were determined with the help of an element analyzer (elementar analysensysteme GmbH - vario EL III). Electrical conductivity for complex **C1** in DMF was measured with a Jenway 4320 conductivity meter. The Fourier transform infrared spectroscopic data for the ligand and its complex pressed in KBr pellets were collected over the range of 400–4000 cm^-1^ on a Nicolet iS10 spectrophotometer. Nuclear magnetic resonance data (^1^H- and ^13^C-NMR) for the yttrium complex in DMSO-d_6_ were recorded with a 400 MHz Bruker NMR spectrometer incorporating tetramethylsilane (TMS) as reference material. The electronic transitions over the 250-800 nm range for the complex were determined with a Perkin Elmer Lambda 40 UV/VIS spectrometer. The X-ray intensity data of complex **C1** were measured at 120(2) K on a Bruker D8 Venture Kappa DUO system equipped with a multilayer mirror monochromator and a MoKα rotating anode X-ray tube (*λ* = 0.71073 Å). The frames were integrated with the Bruker SAINT software package [27]. Data were corrected for absorption effects using the Multi-Scan method (SADABS) [28]. The structure was solved and refined using the Bruker SHELXTL Software Package [29]. All C-bound hydrogen atoms have been calculated in ideal geometry riding on their parent atoms, while the N- and O-bound hydrogen atoms have been refined freely. The crystallographic figures have been drawn with ORTEP-3 at the 50 % ellipsoid probability level [30]. Crystallographic data of complex **C1** have been deposited with the Cambridge Crystallographic Data Centre, CCDC, 12 Union Road, Cambridge CB21EZ, UK. Copies of these data can be obtained free of charge by quoting the depository number CCDC-2117291 (https://www.ccdc.cam.ac.uk/structures/).

### Preparation of the Complex

To a solution of anthranilic acid (274 mg, 2 mmol) in ethanol (100 ml), yttrium chloride hexahydrate (303 mg, 1 mmol) dissolved in a minimum amount of water was added. The solution was boiled for 5 minutes under stirring. After stirring the mixture at room temperature for 1 h, the clear solution was left at room temperature undisturbed for ten days. The single crystals that resulted were filtered, washed with ethanol, and dried in a desiccator over anhydrous calcium chloride.

**[Y**_**2**_ **(HA)**_**6**_ **(H**_**2**_ **O)**_**4**_ **] Cl**_**6**_ **.2C**_**2**_ **H**_**5**_ **OH** (**C1**): White. Yield = 193 mg (42 %). Anal. Calcd. (Found) for Y_2_C_46_H_62_Cl_6_N_6_O_18_ (MW = 1377.54 g/mol): C = 40.11 (40.02) %, H = 4.54 (4.33) % and N = 6.10 (6.68) %. FT-IR (KBr, cm^-1^) = 3360 υ (OH), 1509 υ (COO)_asym_, 1390 υ (COO)_sym_ and 543 υ(Y—O). ^1^HNMR (DMSO-d_6_, 400 MHz) δ/ppm = 8.53 (br, 3H, COO and NH_3_), 7.69 (d, 1H, arom.), 7.21 (t,1H, arom.), 6.74 (d, 1H, arom.), 6.51 (t,1H, arom.), 3.45 (q, 2H, EtOH), 3.33 (s, H_2_O) and 1.06 (t, 3H, EtOH). ^13^CNMR (DMSO-d_6_, 400 MHz) δ/ppm = 18.78, 55.51, 109.04, 114.28, 115.72, 130.63, 133.37, 151.01, and 168.96. Electronic (DMSO, nm) = 338. Molar conductance (DMF, Ω^-1^cm^2^mol^-1^) = 2671.4.

### Evaluation of Cytotoxic Activity on Cancer Cell Lines

MDA-MB-231 human breast cancer cells, PC-3 human prostate cancer cells, and T-24 human transitional cell carcinoma were obtained from the American Tissue Culture Collection (ATCC). The cell lines were cultured in DMEM (Dulbecco’s Modified Eagle Medium) low glucose medium supplemented with 10 % inactivated fetal bovine serum (FBS) and 1 % penicillin/streptomycin prior to incubation in a CO_2_ incubator at 5 % CO_**2**_ and 37 °C. The cell lines were seeded into 96-well plates of 10,000 cells/well. On the following day, the cells were treated with various concentrations (0-1000 μg/ml) of complex **C1** and incubated in a CO_2_ incubator for another 24 h. Indeed, these concentrations were prepared from a stock solution of the complex (100 mg/ml) in DMSO by mixing 0–1 μl from this solution with 100 μl from the growth media. The cell viability was assessed by using MTT (Sigma-Aldrich, St. Louis, MO, USA) by the addition of 10 μl (5 mg/ml) /well before final incubation for 4 h. DMSO (100 μl) was added to each well and the absorbance was read at 570 nm using an ELISA microplate reader (Molecular Devices, Downingtown, PA, USA).

### Statistical Analysis

Data were represented as a mean ± standard error. Data was entered using GraphPad Prism version 5 statistical program (New York, USA). Statistical differences between groups were computed using the one-way analysis of variance (ANOVA) test followed by Tukey’s multiple comparison test. The significant level was set at *P* ≤ 0.05.

## Results and Discussion

### Synthesis and Characterization

The high-purity anthranilic acid ligand (HL) was purchased from Sigma-Aldrich. The synthesis of complex **C1** followed simple procedures as follows. Anthranilic acid (2 × 10^-3^ moles) was dissolved in excess ethanol (100 ml). Afterwards, a millimole of the hexahydrated yttrium chloride salt was dissolved in a minimum amount of water and was added to the ligand solution. Indeed, the reaction medium then is aqueous ethanol. To ensure the reaction completion, we boiled the reaction mixture for 5 min and stirred it for an hour before discarding any solid particles formed in the mixture. The reaction mixture, after ten days, resulted in the recovery of single crystals of complex **C1** in moderate yield (42%). The product was filtered, dried, and exposed to elemental (C, H, and N), spectral (FT-IR, UV-Visible, and ^1^H- and ^13^C-NMR), and crystallographic X-ray diffraction analyses. The complex experienced air stability, light insensitivity, and good solubility in DMF and DMSO. Very interestingly, solution (1 mM) of this complex in DMF afforded a molar conductivity value (2671.4Ω^-1^cm^2^mol^-1^) that is even greater than the reported range for 1:4 electrolytes [31] indicating the very strong electrolytic nature of the complex in solution.

**Fig. 1:**
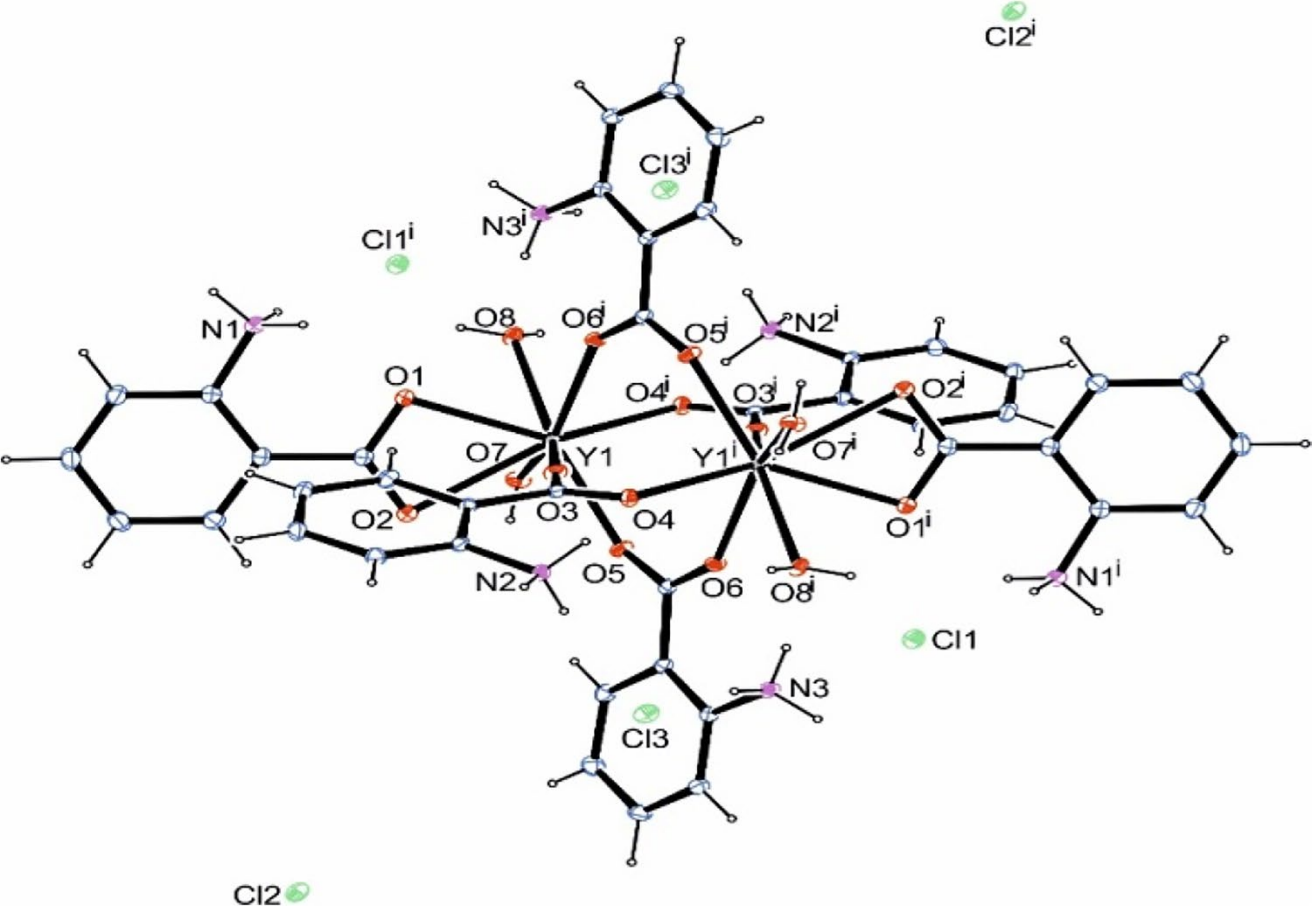
Crystal structure of the yttrium complex. Symmetry code i = 1–x, –y, 1–z

**Fig. 2:**
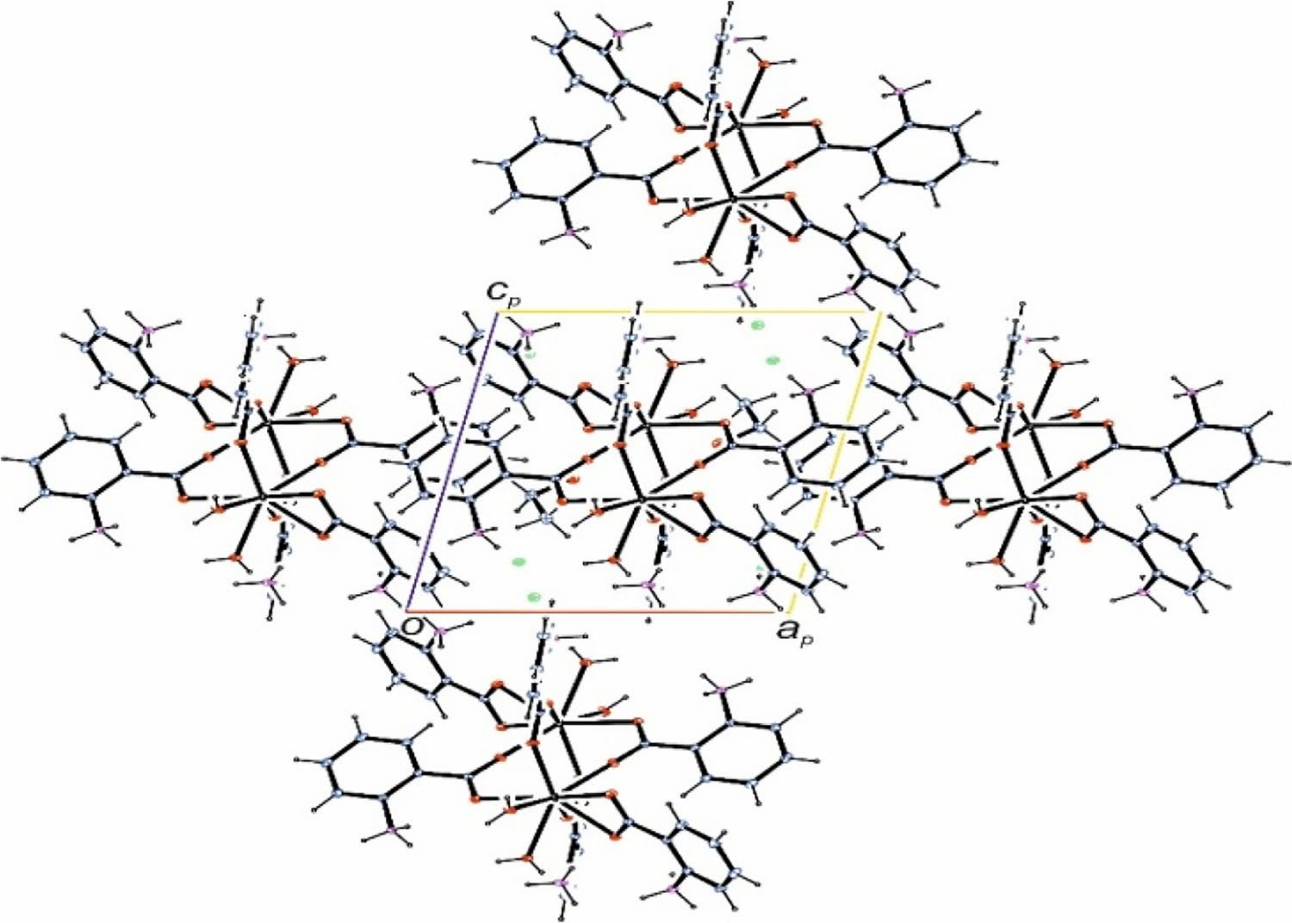
Packing of the yttrium complex viewed along the *b*-axis

The complex CHN data together with the NMR data (see below) confirmed its highest purity as determined by its structure by X-ray single crystal studies. The crystal structure of complex **C1** is shown in Fig. 1 and its packing diagram along the b-axis is given in Fig. 2. Crystallographic and refinement data for the complex are given in Table 1, while selected bond lengths and angles in addition to hydrogen bonding interaction parameters (Table 2) in the complex are listed.

**Table 1:** Crystallographic and refinement data for complex **C1**

Net formula	C_46_H_62_Cl_6_N_6_O_18_Y_2_	Transmission factor range	0.78–0.90
Mr/g mol^-1^	1377.54	Refls. measured	37410
Crystal system	Triclinic	R_int_	0.0201
Space group	P -1	Mean σ(I)/I	0.0140
a/Å	10.8366 (5)	θ range (°)	2.322–27.480
b/Å	11.2950 (6)	Observed refls.	6274
c/Å	12.0567 (6)	x, y (weighting scheme)	0.0227, 0.6186
α/°	80.193 (2)	Refls in refinement	6454
β/°	76.856 (2)	Parameters	409
γ/°	83.077 (2)	R (F_obs_)	0.0175
V/Å^3^	1410.92 (12)	R_w_(F^2^)	0.0455
Z	1	S	1.030
Calc. density/g cm^-3^	1.621	Shift/error_max_	0.001
μ/mm^-1^	2.405	Max, min electron density/e Å^-3^	0.347, −0.214

**Table 2:** Selected bond distances (Å), angles (°), and hydrogen bonding interaction parameters in complex **C1**

Atoms	Bond distance (Ǻ)	Atoms	Angle (°)	
O1—Y1	2.3465 (8)	O1—Y1—O2	53.12 (3)	
O2—Y1	2.5478 (9)	O1—Y1—O3	85.94 (3)	
O3—Y1	2.295 (1)	O1—Y1—O4^i^	138.63 (3)	
O4^i^—Y1	2.3454 (9)	O1—Y1—O5	135.46 (3)	
O5—Y1	2.2917 (8)	O1—Y1—O6^i^	83.60 (3)	
O6^i^—Y1	2.3118 (8)	O1—Y1—O7	94.47 (3)	
O7—Y1	2.366 (1)	O1—Y1—O8	67.76 (3)	
O8—Y1	2.4700 (1)			
Selected data for hydrogen bonding interactions in the complex				
D—H···A	D—H (Ǻ)	H···A (Ǻ)	D···A (Ǻ)	D—H···A (°)
N2—H21···Cl1	0.90 (2)	2.258	3.155	171.61
N2—H22···Cl3	0.90 (2)	2.328	3.197	162.88
N3—H33···Cl3	0.93 (2)	2.314	3.239	170.64
N3—H31···Cl2	0.91 (2)	2.211	3.104	161.61
N1—H12···Cl3	0.93 (2)	2.256	3.177	171.39
N1—H13···Cl1	0.92 (2)	2.213	3.117	168.57
O7—H72···Cl2	0.84 (2)	2.294	3.132	173.64

The XRD analysis of a 0.262 × 0.139 × 0.044 mm single crystal of complex **C1** revealed its dimeric structure and the exhibition of anti-prismatic coordination geometry around each of the yttrium atoms. The yttrium complex crystallized in the triclinic P −1 space group. In this complex, the yttrium atoms are eight-coordinate and all the anthranilic acid ligands are bidentate only via the two oxygen atoms of the carboxylate moiety. However, four out of the six anthranilic acid ligands are bridging the two yttrium atoms resulting in two centrosymmetric eight-membered rings each with a Y_2_C_2_O_4_ set of atoms. Each of the other two 2-aminobenzoic acid ligands is solely bound to one yttrium atom (e.g., Y1 is bound to O1 and O2 from one ligand molecule and O3, O4^i^, O5, and O6^i^ from four anthranilic acid ligands). To complete the coordination sphere, each yttrium atom is further complexed with two oxygen atoms (Y1 is bound to O7 and O8) of water molecules. It should be noted that despite all the ligand carboxylic acid groups being deprotonated, all the anthranilic acid ligands are neutral and exist in zwitter ionic nature due to protonation of the amino groups in the same ligand molecules. Therefore, the charge on the complex ion is compensated only by the presence of six chloride ions out of the complex coordination sphere.

In complex **C1**, a distance of 4.185 Å was detected between the two yttrium atoms. On the other hand, the Y—O (anthranilic acid) distances in complex **C1** range from 2.2917(8) Å (O5—Y1) to 2.5478(9) Å (O2—Y1). These bonds are shorter than bond distances between the yttrium atoms and the coordinated water molecules {2.366(1)–2.4700(1) Å}. It should be noted that the protonated anthranilic acid amine nitrogen atoms do not directly take part in the coordination of the yttrium atoms. However, with other oxygen atoms from water and alcohol molecules, they act as donor atoms establishing an extensive net of hydrogen bonds stabilizing the structure of the complex.

**Fig. 3:**
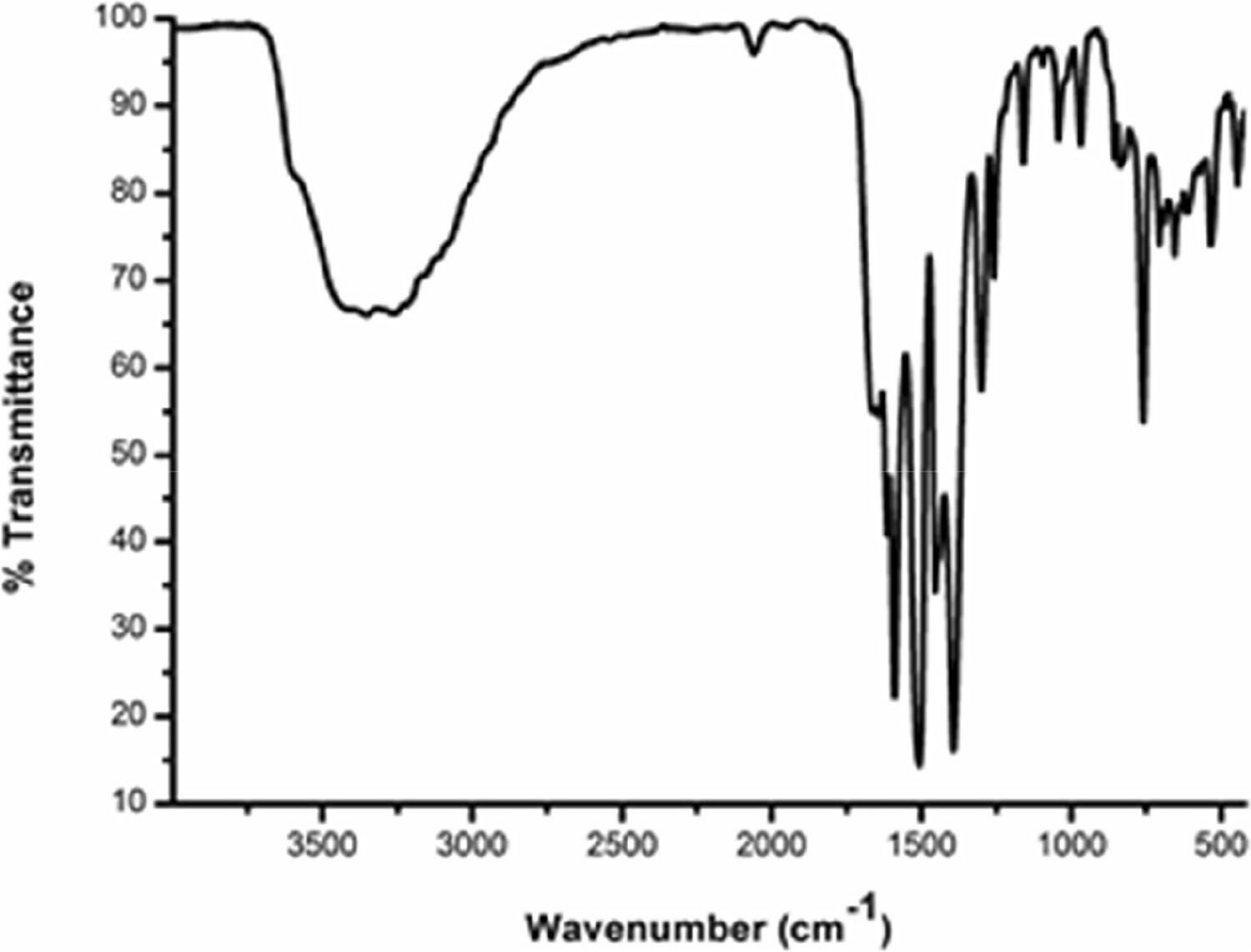
FT-IR spectrum of the yttrium complex

The FT-IR spectral data of complex **C1** (Fig. 3) pressed in a KBr pellet were recorded in comparison to that of the anthranilic acid ligand. In agreement with the literature [20, 32], the free ligand spectrum exhibits a stretching vibration of the OH group at 3584 cm^-1^ in addition to asymmetric and symmetric stretching vibrations of the NH_2_ group respectively at 3376 and 3315 cm^-1^. Moreover, the asymmetric and symmetric stretching vibrations υ (COO) appear respectively at 1670 and 1427 cm^-1^ [20, 32]. Most important in the spectrum of complex **C1**, the asymmetric and symmetric stretching vibrations of the carboxylate (COO^-^) moiety appear respectively at 1509 and 1390 cm^-1^. The wavenumber difference between these band positions indicates the bidentate nature of binding by the carboxylate moiety in complex **C1** [33–36]. Moreover, a new band at 543 cm^-1^ and a broad band at around 3360 cm^-1^ in the spectrum of **C1** are attributed respectively to υ(Y—O) and υ(H_2_O) vibrations in the complex [37–39].

**Fig. 4:**
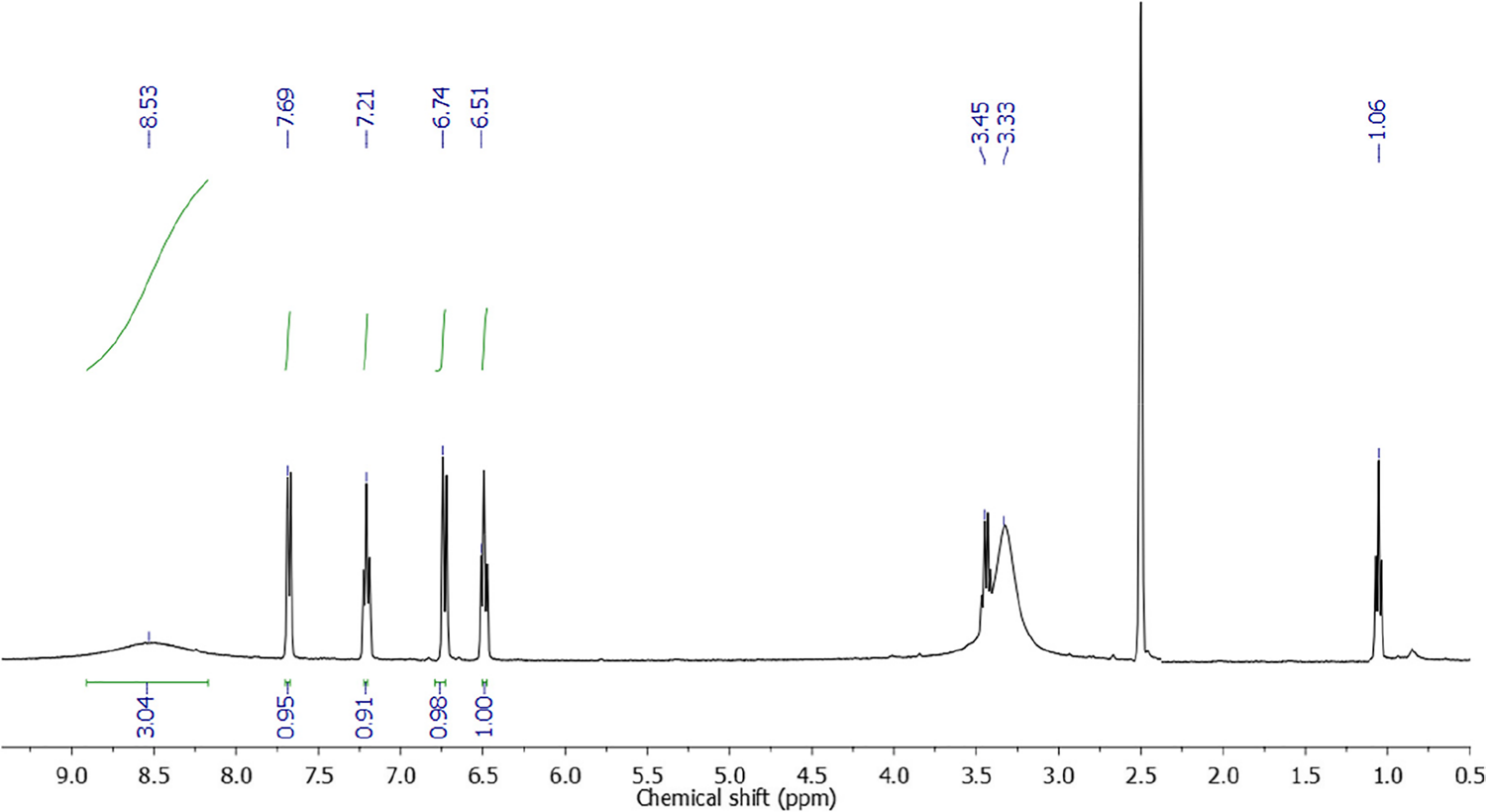
^1^HNMR spectrum of complex C1 measured in DMSO-d_6_

**Fig. 5:**
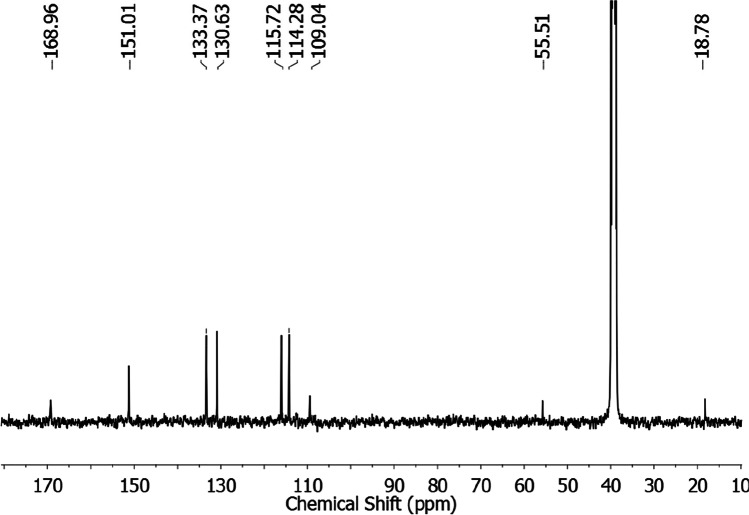
^13^CNMR spectrum of complex C1 measured in DMSO-d_6_

Both ^1^H- and ^13^C-NMR spectra of the complex were recorded in DMSO-d_6_. The complex proton NMR spectrum (Fig. 4) showed no marked difference from that of the ligand [40]. The spectrum displayed four bands over the 6.51–7.69 ppm range attributed to the aromatic ring protons in addition to a broad band present at around 8.53 ppm integrated for the carboxylate group and the protonated amino group hydrogen atoms. The existence of ethanol molecules in the complex was cleared by two bands at 1.06 and 3.45 ppm respectively integrated for 3 and 2 hydrogen atoms. Indeed, these ethanol-attributed bands are present at the same chemical shifts recorded for free ethanol when measured in DMSO-d_6_ [41]. Besides, there should be a band for the —OH moiety of ethanol at 4.32 ppm in the spectrum, but this band is normally broad and was not detected in this study probably due to extreme broadening. Finally, the band present at 3.33 ppm in the complex spectrum is very intense to be integrated for the complex water. This band normally indicates residual water in DMSO-d_6_ solvent [41]. Despite this, we believe this band overlaps with the band for the water molecules in the yttrium complex. On another hand, the yttrium complex ^13^C-NMR spectrum (Fig. 5) demonstrated nine peaks could be correlated due to 2-amibnobenzoic acid (109.04–168.96 ppm [42]) and ethanol (18.78-55.51 ppm [41]) carbon atoms.

### Evaluation of Anti-Proliferative Activities

In this study, we investigated the *in vitro* cytotoxicity of complex **C1** using the MTT assay method against breast (MDA-MB-231), bladder (T-24), and prostate (PC-3) cancer cell lines. These cancer cell lines were treated with various concentrations of the yttrium complex and the dosages that resulted in 50 % inhibition (IC_50_) of the cancer cells were recorded. Indeed, anthranilic acid and many of its derived compounds have been biologically studied for their anti-proliferative activity [3, 43–45]. However, the novelty of yttrium complexes of similar structures used as anticancer agents (in particular against T-24, PC-3, and MDA-MB-231 cells) in the literature made it difficult to sufficiently compare the investigated data for this yttrium complex with other data of similar complexes.

**Fig. 6:**
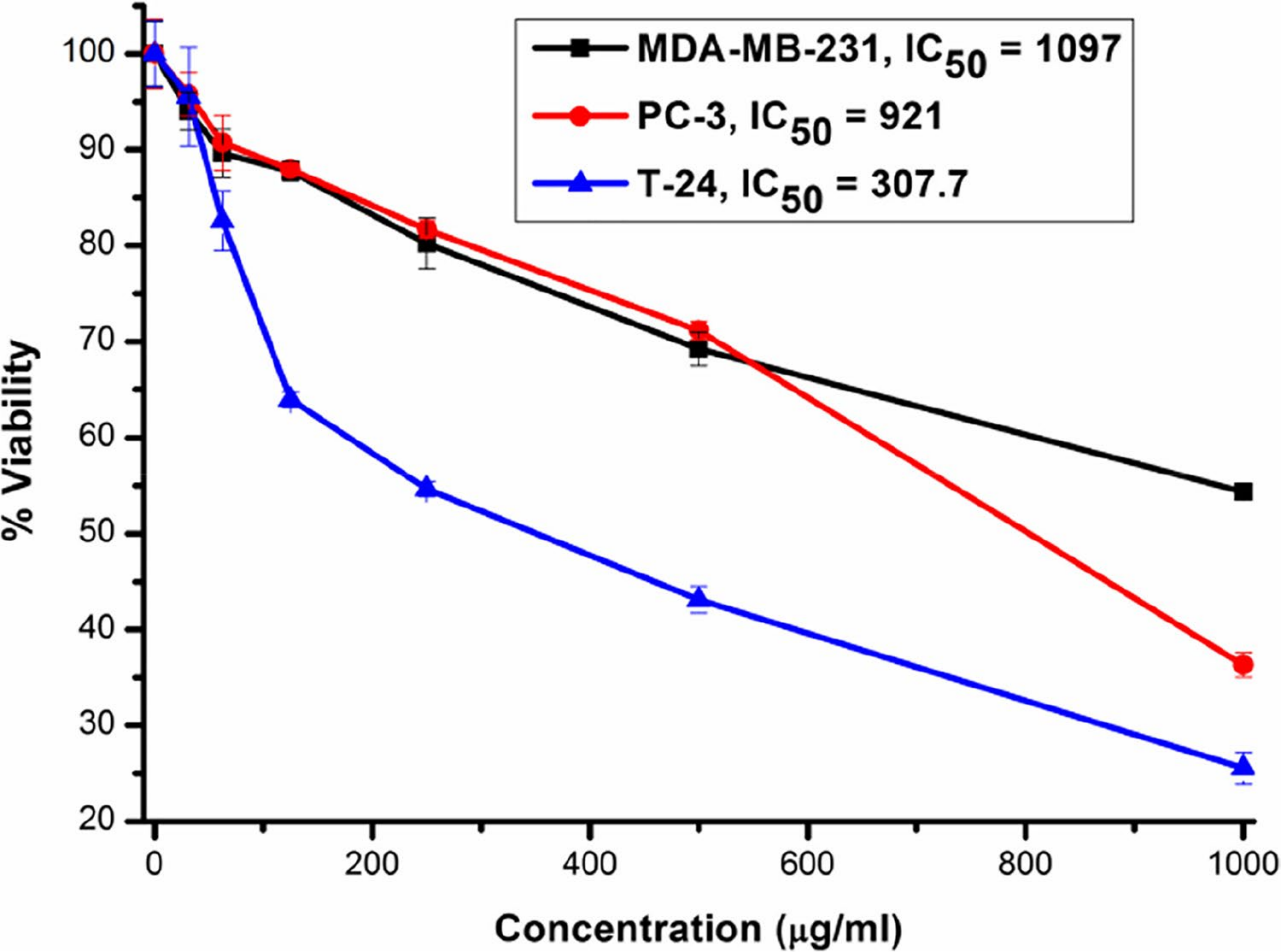
An MTT assay experiment showing the cytotoxic effect of the yttrium complex C1 (0–1 mg/ml) on T-24 (blue), MDA-MB-231 (black), and PC-3 (red) human cancer cells. All data are represented as mean ± SD from three independent experiments and analyzed with two-tailed unpaired Student’s *t*-test. Data is considered statistically significant at (*P ≤ 0.05) and (**P ≤ 0.01)

As displayed in Fig. 6, the yttrium complex showed a moderate cytotoxic effect on T-24 bladder transitional carcinoma with an IC_50_ value of 307.7 μg/ml (223 μM). In addition to that, it exerts a weak cytotoxic effect with IC_50_ {1097 μg/ml (796 μM)} against MDA-MB-231 breast cancer cells and with IC_50_ {921 μg/ml (669 μM)} against PC-3 prostate cancer cells. These results, when compared to obtained data from a recent study on a related binuclear copper complex with a substituted anthranilic acid ligand (i.e., N-acetylanthranilic acid), are much less promising against MDA-MB-231 breast and PC-3 prostatic cancer cells, as the copper complex therein gave IC_50_ values of 131.2 μg/ml (127 μM) and 200.7 μg/ml (195 μM) against these cells, respectively [3]. However, against T-24 bladder cells, the Cu (II) complex gave an IC_50_ value of 300.3 μg/ml (291 μM) which is a comparable value with the IC_50_ value given by the yttrium complex **C1** [3].

Compared with the literature, cisplatin showed great inhibition of MDA-MB-231 cells with IC_50_ values of 1.3 ± 0.55 μg/ml after 24 h and 0.9 ± 0.31 μg/ml after 48 h [46] and the prostatic PC-3 cancer cells died when exposed to cisplatin (22 μM) for 3 days [47]. In turn, the bladder cancer cells T-24 were significantly inhibited by gallic acid that caused inhibitions with IC_50_ values of 21.73, 18.62, and 11.59 μg/ml after 1, 2, and 3 days [48]. Therefore, we conclude that the anti-proliferative activities given by the yttrium complex **C1** against MDA-MB-231, T-24, and PC-3 cell lines are much less significant compared to other compounds and this complex does not appear to represent a promising drug for cancer chemotherapy. However, further research regarding the bioactivity of this complex on animal models or other cell lines may lead to the potential usefulness of it in medicine.

## Conclusions

In this paper, we discussed the synthesis of a new cationic complex of anthranilic acid (HA) {[Y_2_(HA)_6_(H_2_O)_4_] Cl_6_.2C_2_H_5_OH (**C1**)} and determined its exact structure by single crystal XRD analysis that concluded the complex dimeric nature and anti-prismatic geometry in addition to the bidentate nature of the anthranilic acid ligands only via the oxygen atoms. The cytotoxic activity of the complex was studied against three cancer cell lines. Most pronounced was the activity with an IC_50_ value of 307.7 μg/ml displayed by complex **C1** against the bladder cancer cells T-24. However, classical drugs were reported to possess much greater anti-proliferative activity data against MDA-MB-231, T-24, and PC-3 cancer cells in comparison to the yttrium complex in this study.

## Data Availability

Supporting materials will be made available upon request from the corresponding author.
